# Repurposing Oxytocin as A New Predictor for Metabolic Syndrome in Schizophrenia

**DOI:** 10.1192/j.eurpsy.2025.327

**Published:** 2025-08-26

**Authors:** K. K. Goh, M. L. Lu

**Affiliations:** 1 Department of Psychiatry, Wan Fang Hospital; 2 Department of Psychiatry, Taipei Medical University, Taipei, Taiwan, Province of China

## Abstract

**Introduction:**

The increasing prevalence of metabolic syndrome in persons with schizophrenia has driven research into the underlying mechanisms of its pathophysiology. Overlapping neurobiological profiles of metabolic risk factors and psychiatric symptoms, particularly shared genetic liabilities, suggest that oxytocin system dysfunction may be a common mechanism underlying both schizophrenia and metabolic syndrome. Given its prosocial and anorexigenic effects, intranasal oxytocin has been studied for its therapeutic potential in schizophrenia and obesity. However, the pathophysiology and mechanisms of oxytocin in schizophrenia are complex and it is too early to draw definitive conclusions.

**Objectives:**

This study aims to investigate the correlation between plasma oxytocin levels and metabolic syndrome and to test whether oxytocin system dysfunction could serve as a putative endophenotypic marker of schizophrenia.

**Methods:**

This prospective cohort study involved 90 persons with schizophrenia, all of whom were responsive and maintained on stable monotherapy with either olanzapine, risperidone, or aripiprazole, without any alterations to their antipsychotic regimen, alongside 60 healthy controls. All participants were followed for 24 months. Anthropometric measurements, metabolic parameters, and the presence of metabolic syndrome were assessed. Plasma oxytocin levels were measured. The psychopathology of persons with schizophrenia was evaluated using the Positive and Negative Syndrome Scale (PANSS), and quality of life was assessed using the World Health Organization Quality of Life (WHOQOL-BREF) scale. Other potential confounding factors, including dietary habits, caffeine and nicotine use, and menstrual cycle, were considered in the analysis.

**Results:**

Plasma oxytocin levels were inversely associated with the presence of metabolic syndrome in persons with schizophrenia, regardless of antipsychotic treatment. Persons with schizophrenia who had metabolic syndrome at baseline showed lower baseline plasma oxytocin levels. Among those with metabolic syndrome at baseline, higher plasma oxytocin levels were associated with better metabolic parameters at the end of the follow-up period. Conversely, for those without metabolic syndrome at baseline, lower plasma oxytocin levels were predictive of a higher risk of developing metabolic syndrome over time.

**Conclusions:**

This study highlights the potential role of oxytocin in the pathophysiology of metabolic syndrome in schizophrenia. Lower plasma oxytocin levels were associated with both the presence and risk of developing metabolic syndrome, suggesting that oxytocin system dysfunction may contribute to metabolic dysregulation in schizophrenia. These findings support the hypothesis that oxytocin could be a putative endophenotypic marker for metabolic vulnerability in schizophrenia, while future studies should further investigate the therapeutic implications of targeting the oxytocin system.

**Disclosure of Interest:**

None Declared

